# OnGuard3e: A predictive, ecophysiology‐ready tool for gas exchange and photosynthesis research

**DOI:** 10.1111/pce.14674

**Published:** 2023-07-27

**Authors:** Thanh‐Hao Nguyen, Fernanda A. L. Silva‐Alvim, Adrian Hills, Michael R. Blatt

**Affiliations:** ^1^ Laboratory of Plant Physiology and Biophysics University of Glasgow Glasgow UK

**Keywords:** CO_2_, guard cell, humidity, quantitative systems model, stomata, transpiration

## Abstract

Gas exchange across the stomatal pores of leaves is a focal point in studies of plant‐environmental relations. Stomata regulate atmospheric exchange with the inner air spaces of the leaf. They open to allow CO_2_ entry for photosynthesis and close to minimize water loss. Models that focus on the phenomenology of stomatal conductance generally omit the mechanics of the guard cells that regulate the pore aperture. The OnGuard platform fills this gap and offers a truly mechanistic approach with which to analyse stomatal gas exchange, whole‐plant carbon assimilation and water‐use efficiency. Previously, OnGuard required specialist knowledge of membrane transport, signalling and metabolism. Here we introduce OnGuard3e, a software package accessible to ecophysiologists and membrane biologists alike. We provide a brief guide to its use and illustrate how the package can be applied to explore and analyse stomatal conductance, assimilation and water use efficiencies, addressing a range of experimental questions with truly predictive outputs.

## Summary statement

OnGuard3e offers a truly mechanistic approach with which to analyse stomatal gas exchange, whole‐plant carbon assimilation and water‐use efficiency. The software package seamlessly connects whole‐plant gas exchange and photosynthesis with the microscopic mechanics of stomata and is accessible to ecophysiologists and membrane biologists alike. We provide a brief guide to its use and illustrate how the package can be applied to explore and analyse stomatal conductance, assimilation and water use efficiencies, addressing a range of experimental questions with truly predictive outputs.

## 1 INTRODUCTION

Stomata are pores that commonly form between pairs of guard cells on the leaf epidermis of terrestrial plants and enable CO_2_ entry to the leaf for photosynthesis. When open, stomata also provide a pathway for water loss to the atmosphere by evaporation and diffusion of water vapour from the saturated environment of the inner air spaces within the leaf. Guard cells must therefore balance the need for CO_2_ in photosynthesis against the need to prevent drying of the leaf, especially when access to water is limiting. Stomata can reduce photosynthetic rates by 50% or more when limiting water supply to the leaf promotes closure of the aperture and restricts CO_2_ diffusion from the atmosphere (Hetherington & Woodward, 2003; Lawson & Blatt, 2014; Wong et al., 1979). Stomatal behaviour thus encapsulates the tight interdependence between these often conflicting demands.

Research over the past four decades has generated a wealth of knowledge about the molecular, biophysical, and kinetic properties of guard cell membrane transport and metabolism and how they work. Even when removed from the leaf in epidermal peels, guard cells respond in a well−defined manner to an array of extracellular signals, notably light, CO_2_, extracellular solutes and hormones, including the water‐stress signal abscisic acid (ABA) and fungal toxins such as fusicoccin (Blatt & Clint, 1989; Clint & Blatt, 1989). Their ability to regulate stomatal aperture in isolation has greatly facilitated a detailed analysis of the underlying molecular mechanics. From these studies (Jezek & Blatt, 2017; Willmer & Fricker, 1996), we know that guard cells incorporate a number of specialised ion transporters and that they coordinate transport at the plasma membrane and tonoplast through a complex network that spans metabolism, mass action effects and signalling intermediates, to regulate osmotic solute flux, principally of K^+^, Cl^−^ and malate (Mal). These processes, and the accompanying water flux, drive the turgor of the guard cell and stomatal aperture. Such deep knowledge has made the guard cell the single, best‐known cell model for ion transport, homeostasis and signalling.

At the macroscopic scale, stomata connect the global water and carbon cycles, exerting a major influence on both. As one example, foliar transpiration has played an important role in atmospheric modelling and weather prediction for more than a quarter of a century (Beljaars et al., 1996; Berry et al., 2010; Betts et al., 1996; Jasechko et al., 2013). Indeed, stomata have long attracted the interest of modellers seeking to describe their behaviour and the consequences for the plant within mathematical frameworks. These frameworks generally subsume the characteristics of stomata within a small number of empirical parameters that describe stomatal aperture from a phenomenological perspective. They omit the wealth of knowledge, noted above, available for guard cells and the mechanics of how they work. As Berry et al. (2010) pointed out over a decade ago, ‘the decisions “made by” stomata emerge as an important and inadequately understood component of these models'.

With the advent of the OnGuard3 platform, the barriers to such understanding have largely disappeared. The platform seamlessly bridges the gap between the mechanics of the guard cell and the macroscopic frameworks on which much of ecophysiological modelling and analysis have been based (Blatt et al., 2022). As part of its initial unveiling, OnGuard3 exposed a previously unknown and emergent ‘carbon memory’ of stomata: it predicted the slowed the kinetics of stomatal conductance, *g*_s_, that erodes the water‐use efficiency (WUE) under fluctuating light (Jezek et al., 2021). OnGuard3 has since proven an important guide in reverse engineering of guard cells to accelerate responsiveness in whole‐plant *g*_s_ for enhanced WUE and biomass gains (Horaruang et al., 2022). However, effective use of OnGuard3 requires some understanding of ion transport and the principles of membrane biophysics. These are topics that are generally less familiar to most plant biologists, and they present a practical barrier to potential users of the platform.

Here we introduce OnGuard3e for predictive and quantitative analysis of gas exchange physiology. Like the OnGuard3 platform (Jezek et al., 2021), OnGuard3e offers a mathematical framework for modelling and analysis of plant‐atmospheric interactions, and it incorporates the depth of mechanistic knowledge available for guard cell transport, metabolism and signalling. However, OnGuard3e places the molecular mechanics of stomata within an ecophysiology‐ready format that should ease its use in many settings.

## 2 INSIDE THE OnGuard3e PLATFORM

**Figure 1:**
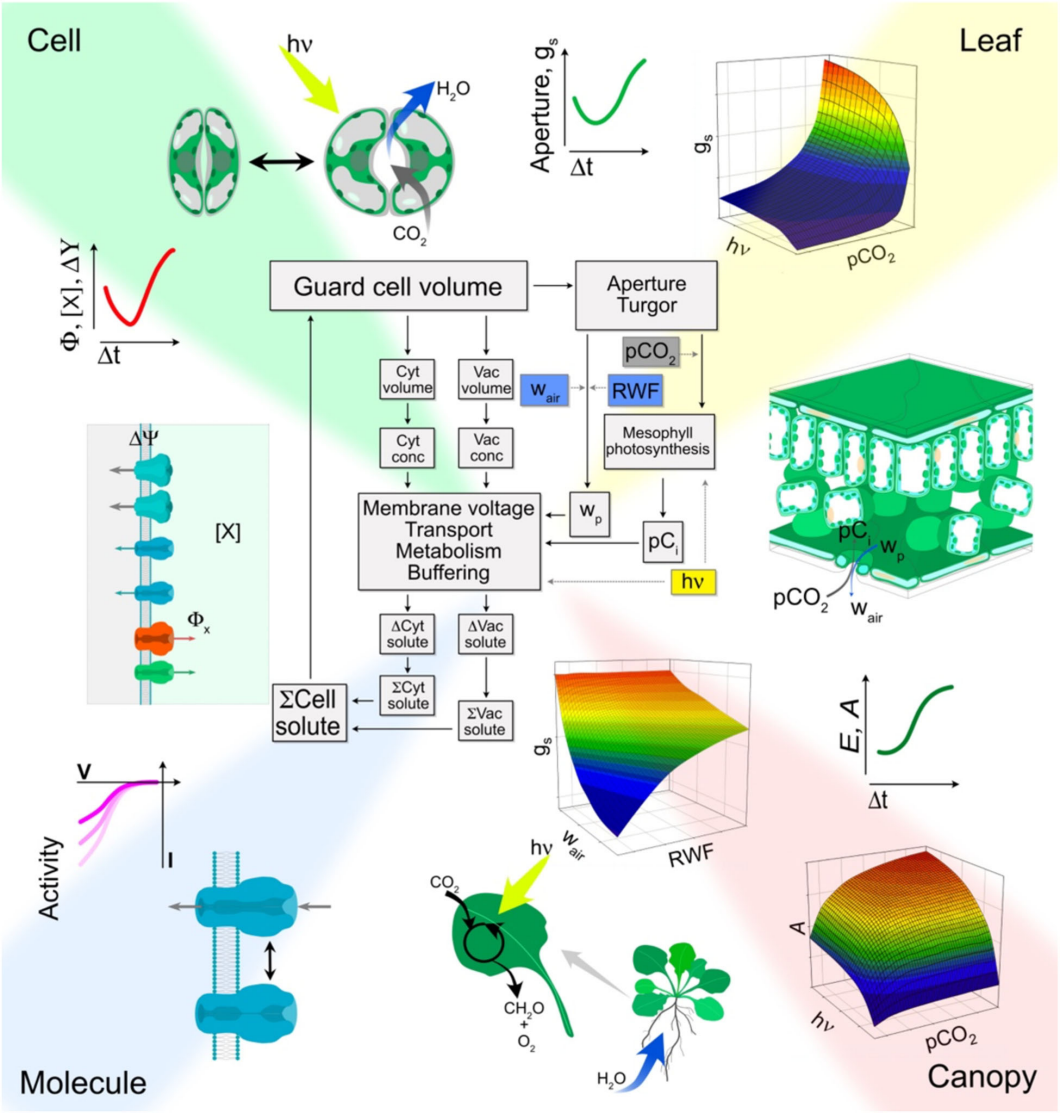
OnGuard3e gives access to a wide range of outputs from the temporal kinetics of the cell to whole‐plant gas exchange and photosynthesis. The computational cycle (center) of the OnGuard3e platform is shown surrounded by the range of output groupings that the platform makes available to the user. These groupings cross scales from the activities of single ion channels to assimilation (*A*), transpiration (*E*) and stomatal conductance (*g*_s_) of the whole plant. OnGuard3e computes the changes in solute gas and metabolic flux, clockwise over the cycle, with small increments in time (Δ*t*) as indicated by the black arrows within the cycle. Environmental inputs comprise light (*hν*), the relative water feed (RWF) that subsumes water flux and its availability to the leaf, and the atmospheric partial pressures of water vapour (*w*_air_) and CO_2_ (pCO_2_), each indicated by the coloured boxes and grey arrows. Model outputs evolve with each increment in time and distribute between the molecular, cellular, leaf and canopy. Molecular and cellular outputs, indicated within *Molecule* and *Cell* quadrants, include the activities of transport and metabolic reactions, here illustrated with current–voltage curves; the cytosolic and vacuolar solute contents ([X]); the compartmental metabolic and solute fluxes (*Φ*_X_); and the voltages (Δ*Ψ*) across the plasma membrane and tonoplast. The guard cell osmotic potential, volume and turgor, and hence stomatal aperture and conductance of *Cell* and *Leaf* quadrants, are derived from these outputs. Foliar outputs and the steady‐state surface plots of *Leaf* and *Canopy* quadrants are determined by the cellular outputs together with the environmental inputs of RWF, light, *w*_air_ and pCO_2_, and the intermediates internal to the leaf, namely the partial pressures of water vapour (*w*_p_) and CO_2_ (pC_i_). Phenomenological models address only the *Leaf* and *Canopy* characteristics and lack mechanistic connection to the *Molecule* and *Cell* processes of the left side of the figure.

The engine behind OnGuard3e is identical to that of the full OnGuard platform (Chen et al., 2012; Hills et al., 2012; Jezek et al., 2019, 2021; Wang et al., 2017) and incorporates the mechanics of solute transport, buffering and metabolism in the guard cells. Within the core of the platform, all metabolic and transport processes and their regulatory properties are fully defined by standard, nonlinear differential equations with parameters determined experimentally. OnGuard3e uses the same iterative computational cycle to introduce deviations away from the previous steady state with small steps in time. The platform calculates and logs the dynamic adjustments of solute flux, compartmental composition, and voltage across the guard cell plasma membrane and tonoplast (Figure 1). All of these calculations are constrained by fundamental physical laws of mass and charge conservation, and they generate new solute contents for the guard cell compartments of the cytosol and vacuole at the end of each time interval. In turn, these values are used to calculate the new total and compartmental cell volumes, solute concentrations, guard cell turgor, and stomatal aperture, as well as solute exchanges with the neighbouring epidermal cells. Thus, rather than transiting between predefined limits, OnGuard3e outputs *evolve* over time, just as stomatal apertures do in vivo, determined by the mechanisms encoded in the metabolic and transport flux equations, the variables they generate, and the network of interactions between these processes engendered by the variables and their entanglements.

In its original formulation (Chen et al., 2012; Hills et al., 2012), the OnGuard platform described stomata as if isolated in epidermal peels. Models built on this platform nonetheless provided reasonable simulations of stomatal dynamics and uncovered an emergent network connecting the KAT1 K^+^ channel with the SLAC1 anion channel (Wang et al., 2012) as well as explaining the origins and impacts of oscillations in cytosolic‐free Ca^2+^ concentrations ([Ca^2+^]_i_) (Minguet‐Parramona et al., 2016). OnGuard2 introduced the hydraulic feed of the whole plant, foliar transpiration and water transport in the guard cells (Wang et al., 2017). It used vapour equilibration with the water potential in the guard cell wall to harmonise the platform with concepts of liquid water delivery to the mesophyll and guard cells (Buckley et al., 2017, 2003; Peak & Mott, 2011; Rockwell et al., 2014). Apoplastic solute and turgor ‘exchange’ with the surrounding epidermal cells was introduced to accommodate these opposing mechanical pressures of the epidermis and the viscoelastic properties of the guard cell wall on stomatal aperture (Jezek et al., 2019). Finally, OnGuard3 added CO_2_ diffusion from the atmosphere and its fixation through mesophyll photosynthesis (Jezek et al., 2021). Adding CO_2_ diffusion to the platform allowed for model constructs that tested the minimum set of targets necessary for CO_2_ to act on stomatal aperture. It uncovered an emergent ‘carbon memory’ in stomatal responsiveness that was predicted, and subsequently shown, to erode *g*_s_ kinetics and the WUE of plants under fluctuating light and pCO_2_ (Jezek et al., 2021). Most recently, OnGuard3 was used to guide the engineering of a guard cell ion channel for improved WUE and biomass gains (Horaruang et al., 2022).

Outputs generated by OnGuard3e, as with OnGuard3, capture all of the temporal kinetics of the guard cells, second by second, as the plant trades soil water for atmospheric CO_2_. These outputs include the osmotic solute content, turgor and volume of the guard cell, stomatal aperture and conductance (*g*_s_), and the rates of transpiration (*E*) and carbon assimilation (*A*), all as functions of light, atmospheric CO_2_ (pCO_2_), and relative humidity (RH) as well as water and solute availability. OnGuard outputs also encompass the subcellular kinetics of solute flux and the molecular activities of individual guard cell transporters, and all of these model components and their associated variables are available for analysis (Figure 1).

**Figure 2:**
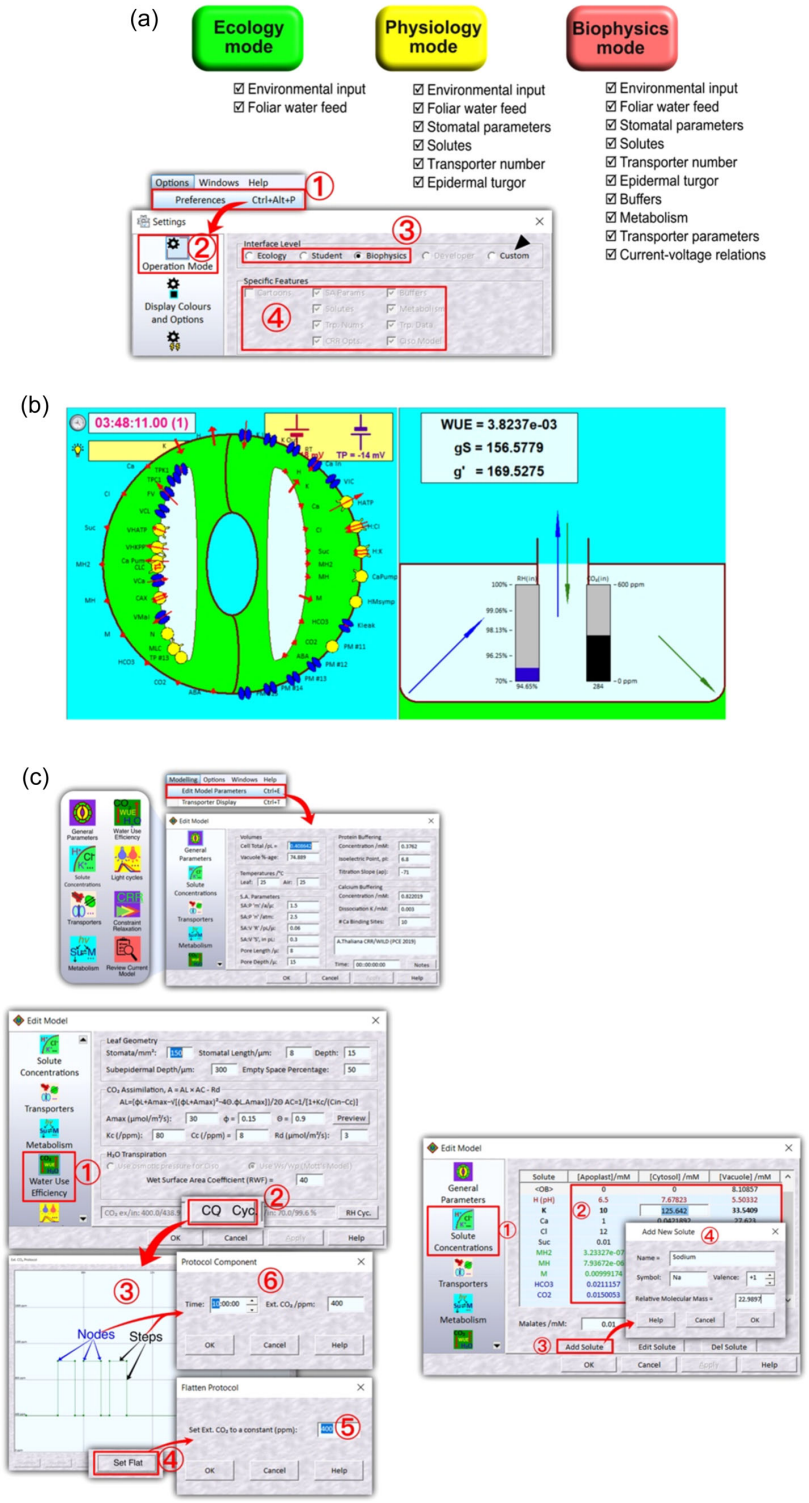
OnGuard3e working modes and model parameter access. (a) OnGuard3e provides three working modes, *Ecology, Phyiology* and *Biophysics*, with increasing access rights to the parameters defining a model. Each mode is designed to fit the respective user expertise. To choose the appropriate working mode, go to *Options*, *Preferences* from the dropdown menu or press *Ctrl* + *Alt* + *P* (1). From the *Settings* window, click on the *Operation Mode* button (2) on the left side, and select the appropriate mode in the *Interface Level* field (3). The information about the features provided by each mode is presented in the *Specific Features* field (4). Users cannot add or remove these features unless the *Custom* mode (black arrow) is chosen in the *Interface Level* field. (b) OnGuard3e includes optional cartoon displays that are updated during simulations when run in the *Begin Simulation* mode. Shown here are the transport (*left*) and gas exchange (*right*) displays. The transport display shows the ion fluxes across each of the two membranes, both as total flux for each ionic species and as the flux through the individual transporters. The gas exchange display shows the net water vapour (*blue*) and CO_2_ (*green*) fluxes through the stomatal pore (*center*) and within the leaf (*corners, left* and *right*). Also shown is the current relative humidity and CO_2_ partial pressure within the leaf air space. Arrows in every case are scaled logarithmically so that a twofold change in length indicates a 10‐fold change in flux. (c) Model parameters are divided between eight property pages in the *Edit Model* window, accessed by going to *Modelling, Edit Model Parameters* from the dropdown menue or pressing *Ctrl* + *E*. In the *Edit Model* window, the icons representing the eight property pages are shown on the left (*upper panel*). The information and adjustable parameters relating to each property are shown in each of the main windows (*upper panel right* and *below*). The *Water‐Use Efficiency* page with changes to the CO_2_ cycle are shown (*below, left*) as an example of modifying cycle‐based model parameters. The *Ext. CO_2_ Protocol* pop‐up window, which is accessed by following steps (1) and (2), provides the adjustable CO_2_ cycle (3), the option to set a constant external CO_2_ value with the *Set Flat* button (4) of a given value (5). Protocols with CO_2_ changes during the day can be achieved by repositioning nodes (blue arrows) and steps (black arrows) or by double‐clicking and entering the precise time point and CO_2_ value (6). Nodes can be added at the cursor location or selected and removed from the protocol by clicking *New Corner* or *Delete Hook*, respectively. OnGuard3e allows users to control the daily cycle of four environmental inputs, atmospheric CO_2_ and relative humidity (RH), blue and red light. Light is accessed through the *Light Cycles* page and can be modified similarly. The *Solute Concentrations* (1) page (*bottom, right*) gives the user access to parameters relevant to guard cell solute flux. In the *Physiology* and *Biophysics* modes, the concentration of each solute in each compartment is accessible by clicking directly on the value and typing a desired number (2). In the *Biophysics* mode, new solutes can be added to the model by clicking the *Add Solute* button (3) and subsequently entering all required information into the pop‐up window (4). Users can also change the information of a solute via *Edit Solute* and remove a solute by clicking *Del Solute*. It is advisable, when adding and deleting solutes—as when altering solute concentrations—to ensure any changes are balanced in charge. Note that adding a new solute will not impact on model outputs unless an appropriate set of transporters at each membrane are also added to the model. Likewise, entirely removing a solute from a model without also removing the associated transporters is guaranteed to cause computational failure. In the *Ecology* mode, access is restricted to the concentrations of extant solutes in the apoplast.

**Figure 3:**
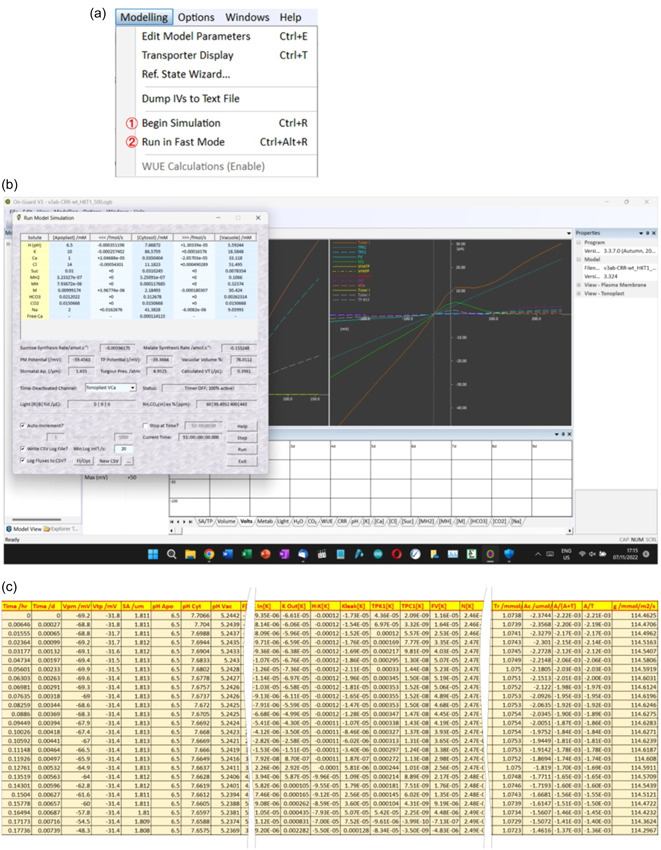
Running and logging an OnGuard simulation. OnGuard simulations are run from the *Modelling* dropdown menu (a) using either the *Begin Simulation* (1) or the *Run in Fast Mode* (2) command. The *Begin Simulation* command opens a flux window (b) that also gives the user options for time autoincrements and CSV file logging, as well as readouts for the fluxes across the plasma membrane and tonoplast and contents of the major compartments of the guard cell. CSV files (c) can be selected to log both net fluxes as well as the individual ion and solute fluxes along with a full range of intermediate and macroscopic variables with each time increment.

Indeed, previous publications offer many examples of ecophysiologically focused outputs from OnGuard. Among these, examples will be found in figures 2, 3, 5–7 and Supporting Information figure 1 of Wang et al. (2017) of how the platform predicts *g*_s_ and associated variables in response to the atmospheric partial pressure of water vapour (*w*_air_) and the vapour pressure difference, to the water feed to the leaf (RWF), and to leaf temperature. Examples of OnGuard predictions for *g*_s_, *E* and *A* in response to opposing changes in light and atmospheric partial pressure of CO_2_ (pCO_2_) are presented in figure 2 of Blatt et al. (2022). OnGuard model predictions for *g*_s_, *E*, *A*, the associated cellular variables of ion flux in response to light, atmospheric CO_2_, and their oscillations will be found in figures 2 and 5 and Supporting Information figures S2, S3 and S7 of Jezek et al. (2021). Finally, examples of *g*_s_ and its kinetics predicted for modified GORK K^+^ channels will be found in figure 5 of Horaruang et al. (2022). Each of these publications also includes experimental validations of the key OnGuard predictions, demonstrating how the platform seamlessly connects the macroscopic outputs of gas exchange with the microscopic characteristics of guard cell ion and water flux.

Users of OnGuard3e are likely to be interested also in a number of intermediate variables. For example, the platform gives access to the relative humidity (RH_i_), or partial pressure of water vapour (*w*_p_), and CO_2_ (pC_i_) within the leaf air space of the substomatal cavity, as well as the concentrations of all of the major solutes and signalling intermediates in the major compartments of the guard cell (Blatt et al., 2022; Jezek et al., 2021; Wang et al., 2017). Each of these variables is connected to *w*_air_ and pCO_2_, the fluence rate of light, and the rate of mesophyll carbon assimilation. Finally, all OnGuard3e outputs take the form of real−time temporal kinetics much as would be recorded in experiments, whether of whole‐plant gas exchange or of single‐cell physiology with guard cells in isolation.

## 3 PHILOSOPHY AND LIMITATIONS

It is the gold standard in modelling to ask ‘What is the most parsimonious assembly of model elements needed to simulate the biological system?’ Beyond parsimony, useful models not only recapitulate known behaviours, they also predict new ones. Thus, the modeller will also ask ‘What behaviours does this assembly predict that are experimentally testable?’ Equally informative, the modeller will want to know ‘In what ways does the model assembly fail, either in recapitulating known behaviours or in predicting new ones?’ In short, each OnGuard model—each *.ogb file—becomes a hypothesis under test, to be discarded, validated, or refined by comparisons between model predictions and experimental results. Because the kinetics of most biological processes are highly nonlinear, often with respect to multiple parameters, the interactions of these processes underpin much of physiology that is seemingly counterintuitive, what are often termed ‘emergent’ behaviours. More often than not, these emergent behaviours become a focus for model testing and validation.

OnGuard facilitates modelling of this kind, building on the depth of quantitative knowledge outlined above and detailed previously (Chen et al., 2012; Hills et al., 2012; Jezek et al., 2021; Wang et al., 2017). The parameter space for guard cell transport and metabolism is exceptionally well defined experimentally, generally to within a factor of three and frequently with an accuracy of a few percent (Hills et al., 2012), and similar degree of accuracy is evident in the macroscopic characteristics pertaining to gas exchange for a wide range of species (Leakey et al., 2019; Long et al., 2022; Matthews & Lawson, 2019). This knowledge, and the fundamental requirements for charge and ionic balance, constrain the few model components for which parameterization is less well−defined.

Of course, as with any mechanistic modeling, success with the platform rests with the ability of the modeller to adequately populate the parameter set—the *.ogb file—that defines a model. For now, the OnGuard platform works well for a range of kidney‐shaped stomata. Models for *Arabidopsis*, *Vicia* and tobacco are available for download; extensive descriptions of the parameter sets and their justifications will be found in Hills et al. (2012), Chen et al. (2012), Jezek and Blatt (2017) and Jezek et al. (2021). Of course, these models come with the standard proviso of all working systems: they are good approximations to experimental data within the bounds of the conditions and data used for their validation. With new experimental data, further refinement of one or more model parameters may be needed in the future. We welcome communication with users and are always open to suggestions for refinements and new implementations to the OnGuard platform.

With minor parameter adjustments, the *Arabidopsis* model will accommodate the behaviours of other *Brassica* species, and close relatives *Tarenaya hassleriana*, and the C_4_ model *Gynandropsis gynandra* (Alvim, 2022). Likewise, the *Vicia* and tobacco model requires little adjustment to accommodate the behaviours of potato, tomato and pea. We are presently developing the parameter set for *Zea mays* for which some essential detail of the stomatal complex is available (Büchsenschütz et al., 2005; Fairley & Assmann, 1991; Fairley‐Grenot & Assmann, 1992; Majore et al., 2002). Otherwise, there is far less quantitative data suitable for modelling dumbell−shaped stomata, which is likely to limit applications of the OnGuard platform with many grasses for now.

There remain other important gaps in our knowledge of stomata that users need to be aware of, although many are accommodated within parameter assignments. Users will find extensive discussions of these gaps in relation to the OnGuard platform in several of our previous publications (Blatt et al., 2022; Chen et al., 2012; Hills et al., 2012; Jezek et al., 2019, 2021; Vialet‐Chabrand et al., 2017; Wang et al., 2017). Among these, are the kinetic relationships of (de)phosphorylation and impacts on organic solute metabolism ion channel activities (Lefoulon, 2021). For example, we know that [Ca^2+^]_i_ inactivates inward‐rectifying K^+^ channels, possibly through the actions of one or more Ca^2+^‐dependent protein kinases (Acharya et al., 2013; Li et al., 1998; Ronzier et al., 2014; Zou et al., 2015). We lack the detail needed to model Ca^2+^ binding and channel phosphorylation. Nevertheless, we know the overarching kinetics of [Ca^2+^]_i_ action on K^+^ channel activity and can place the intermediate steps within a mathematical description that subsumes these details. This approach introduces modules—effectively ‘black boxes’ with adjustable levels of resolution—that may be expanded if, and when, the detail of a specific module becomes a focus; it also greatly reduces the computational overhead of the model without loss of predictive power (Endy & Brent, 2001). There are many successful examples of black‐boxing in biology, including the Hodgkin–Huxley equations used to represent the Na^+^ and K^+^ channels now known to drive the action potentials of nerve and muscle. Indeed, the Hodgkin–Huxley equations explained the fundamental physiological processes of channel gating decades before the underlying molecular mechanisms were elucidated (Hille, 2001).

Finally, it is important to note that each OnGuard model builds on a single set of parameters and therefore represents the ‘ideal’ or averaged behaviour of stomata behind foliar gas exchange. Experimental strategies that depend on selecting individual stomata, subsets of stomata over the leaf surface, individual leaves or plants invariably yield results that are biological snapshots. These snapshots are governed by heterogeneities in the underlying processes that may vary, each within a normal distribution of values. From a modelling standpoint, it is common practice to explore the range of population behaviours by introducing meaningful variations in combinations of parameters to acertain the range of outputs that arise from interactions intrinsic to the model. These outputs can then be compared with the experimental results and with the data typical from the species; they also provide important insights into the sensitivities of a model to specific parameter choices (Hills et al., 2012; Vialet‐Chabrand et al., 2017).

## 4 RUNNING OnGuard3e

OnGuard3e is available as a standalone package and is compatible with all currently supported Microsoft Windows operating systems. Versions are available for 32‐ and 64‐bit platforms and will also run on a Mac using a Windows ‘emulator’ such as Parallels or the native Windows mode of Boot Camp. The 32‐bit version of OnGuard3e also works on Mac computers with the M1, M1X, M2 and M2X chips. The software can be downloaded and installed using the relevant (32‐ or 64‐bit) msi installer file and includes an extensive set of guides and context‐sensitive help pointers, the latter accessed by hovering over the various parameter controls and buttons.

OnGuard3e uses the standard file dropdown structure familiar to Windows users. It runs in three, main, user‐selectable modes that are accessed in *Preferences* from the *Options* dropdown menu (Figure 2a). The user has the option to switch between modes at any time, including during a modelling session. The *Ecology* mode operates with access to environmental inputs and water feed only and is suitable for simulations and analyses that explore these inputs with species compatible with any of the established models supplied with the platform. Additionally, the *Ecology* mode includes intuitive, animated displays of real−time water and CO_2_ fluxes, the RH and partial pressures of CO_2_ in the atmosphere and intercellular leaf air space, the rate of carbon assimilation, and macroscopic properties of the guard cells and stoma (Figure 2b). The *Physiology* mode gives access to a more extensive subset of model parameters, including all solute concentrations, some properties of the membrane transporters, guard cell metabolism, the relationship between guard cell solute content, volume, turgor and aperture, and parameters defining ion and turgor ‘exchange’ with the surrounding epidermal cells. The *Physiology* mode is likely to attract researchers working in ecophysiology, not least because it is possible to extend existing models to accommodate new species of interest. The *Biophysics* mode gives full access to all OnGuard controls and displays, including transport current–voltage relations. Both the *Physiology* and *Biophysics* modes include real−time, animations describing the ion fluxes through each membrane and transporter. The *Biophysics* mode can also be used to define entirely new parameters for species that have yet to be modelled as well as introducing new ion transport pathways, for example to explore the effects of salinity stress on stomatal function (Nguyen et al., 2022). It is most suited to researchers working in fields close to membrane biology.

Regardless of the mode selected at startup, we recommend opening one of the model files (*.ogb, located in the default folder, created on installation) supplied with the platform. Once open, the file will populate the full set of parameters along with settings for the diurnal pattern of light, pCO_2_, *w*_air_, and hydraulic conductance encapsulated in the relative water feed (RWF). Simulations may be run using the *Begin simulation* command, which opens the flux window, or through the *Run Fast* mode command, both found in the *Modelling* dropdown tab (Figure 3a). Simulations that run in the *Begin simulation* mode will update the animations and displays, including a flux window (Figure 3b), throughout and give the user access to a number of controls while in simulation. The *Run Fast* mode limits user access and the real‐time display during simulations, and it does not support animations, but it is substantially faster in operation. Both simulation modes will generate a complete set of outputs in spreadsheet‐readable (*.csv) format (Figure 3c) that are then available for import into a number of analysis and graphical display programs (Chen et al., 2012; Hills et al., 2012; Jezek et al., 2021; Wang et al., 2017).

From the Edit Model Parameters command in the *Modelling* dropdown of OnGuard3e, the user has access to a range of environmental and associated parameters (Figure 2c). For example, if you have opened the ARAB‐wt.ogb file, then in the Model View panel *Water‐Use Efficiency* a double‐click on *CO_2_ cyc* will open a pop‐up window to show a 24 h diurnal cycle preset with 1‐h steps from 400 to 1000 ppm separated by periods at 400 ppm CO_2_ (Figure 2c). To remove the steps, click the *Set Flat* button, enter the desired pCO_2_, click ‘OK’ and then ‘OK’ again to exit the CO_2_ cycle editor. Steps in pCO_2_ may also be introduced by clicking on an interval between nodes in the window and then dragging the interval to the desired pCO_2_ value or by double‐clicking and entering a numerical value. Nodes and steps can also be repositioned by clicking and dragging or by numerical entry.

A similar set of user‐accessible controls are available for the diurnal cycles in RH (*w*_air_) and in light. OnGuard3e users have access also to define apoplastic ion and other solute concentrations, ABA (see below), fluence rates of blue and red light, pCO_2_, water availability (as RWF), settings for mesophyll photosynthesis, leaf geometry, air and leaf temperatures. It is possible to reset operational variables, such as the elapsed time, and to save and/or rename a model file. Be aware that saving an open model will overwrite the existing *.ogb file, unless the Save As… option is selected from the *File* menu. Saving a model also saves the current simulation timepoint, which can cause confusion when running the model afresh after saving and exiting OnGuard3e unless the elapsed time is first reset to zero. A brief summary of the model elements are outlined in the next section.

Model parameters cannot be changed while a simulation is running. However, the user can pause a simulation at any time and then resume the simulation when ready. This facility is particularly useful as it allows the user to modify one or more model parameters while the simulation is paused to test the consequences of the modifications over time when the simulation resumes. The user can also program the simulation to pause at a preset time. Thus, it is possible to simulate changes in any number of environmental and endogenous stimuli at a specified time in the diurnal cycle and to follow the progression of changes in stomatal response and its impact on *g*_s_, *A* and *E*, among other outputs.

**Figure 4:**
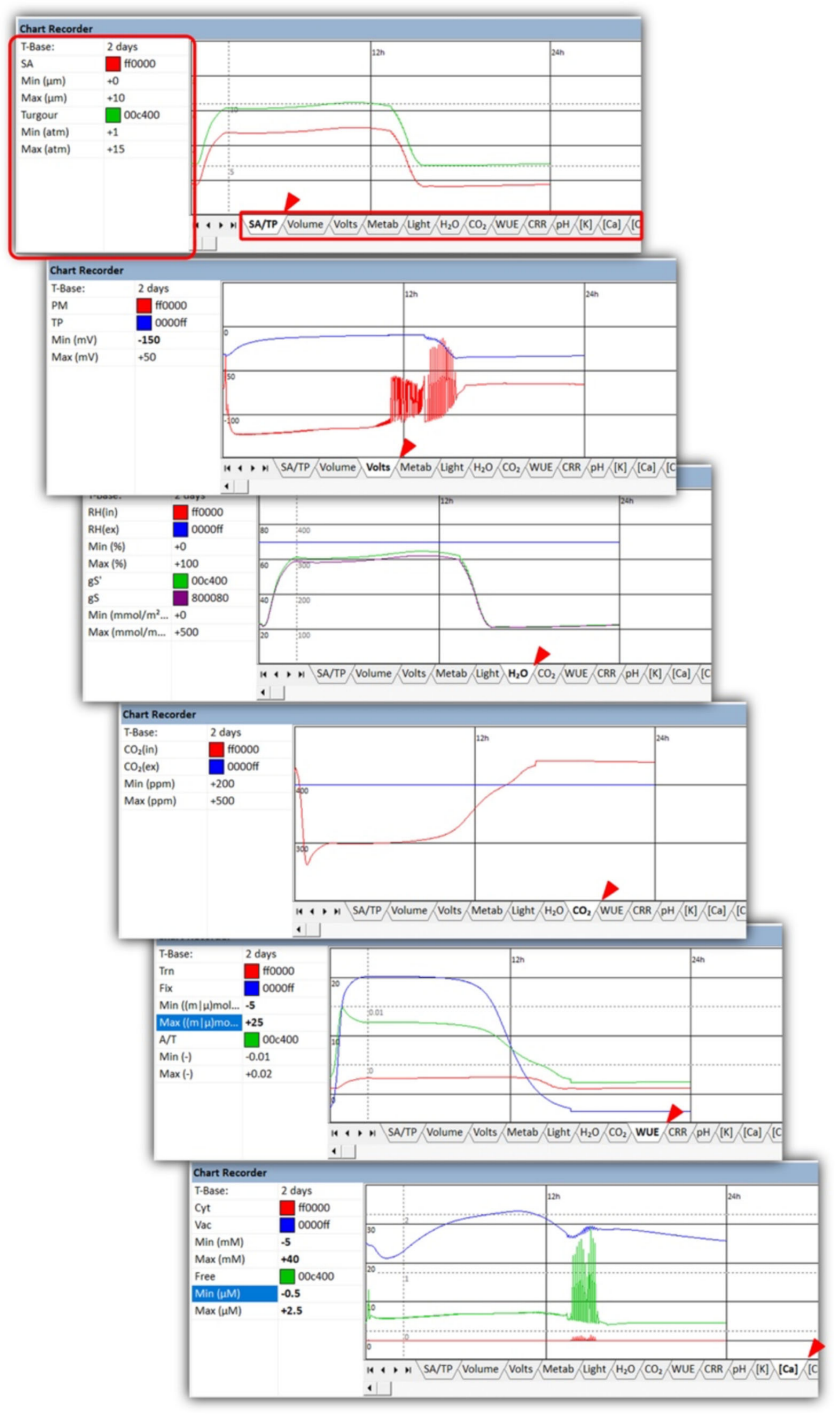
Real‐time tracking of a simulation using the Chart Recorder. The *Chart Recorder* tool of Onguard3e provides real‐time visualization of a selection of commonly sought outputs during the simulation. The simulation mode (*Begin Simulation* or *Run Fast* mode) determines how the user interacts with *Chart Recorder*. In *Begin Simulation* mode, the user can switch freely between *Chart Recorder* tabs (*red box and ticks*) during simulation to observe the changes of different parameters, adjust the time base for all tabs, and adjust the scaling within each tab. In *Run Fast* mode, the time base and scaling are not accessible during simulation and switching between tabs must be done using the drop‐down list within the *Run Fast* mode box. The appearance of the chart recorder can be customized by modifying the parameters in the chart properties table when a simulation is not running, or is paused. Adding a new solute generates a new chart recorder tab once the model is saved and reopened.

In all operational modes, OnGuard3e provides a real‐time, chart recorder onscreen for many outputs (Figure 4) in addition to the full set of outputs logged to a spreadsheet‐readable (*.csv) format. Chart recorder data are also saved automatically as OnGuard *.ogc files that are available for later review. A typical 24‐h simulation started with *Begin simulation* will complete within 10–20 min on a quad‐core i7 computer, depending on the environmental steps included, for example in pCO_2_ and *w*_air_. The same 24‐h simulation run in the *Run Fast* mode will usually complete within 3–5 min. It is possible also to shift between these two simulation approaches when a simulation is paused.

## 5 MODEL ELEMENTS

Much of the OnGuard platform includes parameters that are scaled to the guard cell, reflecting the fact that the vast majority of stomatal research at the cellular level is carried out on guard cells isolated in epidermal peels, or as protoplasts and isolated vacuoles, maintained in controlled external media. Parameters and variables relevant to macroscopic outputs are scaled to the leaf surface area. Sugar and Mal synthesis in the guard cell (see metabolism, Figure 2c), for example, are defined as rates per guard cell in units of fmol s^−1^, whereas macroscopic outputs are defined per unit leaf surface area. Thus, carbon assimilation is defined in units of μmol m^−2^ s^−1^ and transpiration is defined in units of mmol m^−2^ s^−1^. Of course, knowing the stomatal density over the leaf surface, it is a straightforward matter to convert between these two sets of parameters. *General Parameters* (Figure 2c) relate to the guard cell volume, turgor and aperture relations, temperatures and elapsed time. The *Solute Concentrations* page (Figure 2c) reports on the solutes in each of the three compartments associated with the guard cell, namely the apoplast, cytosol and vacuole; it gives the user access to modify the apoplast composition; in the *Physiology* mode the user can modify all solute compositions; and in the *Biophysics* mode it is possible to introduce new solutes as well.

The OnGuard platform treats the bulk apoplast surrounding the guard cells as a reservoir that is unaffected by solute taken up or released by the guard cell; its composition is defined therefore by the user. However, solute gain and loss from the guard cell is connected with solute loss and gain by the epidermal cell immediately adjacent the guard cell, in effect creating a subdomain of the apoplast between these two cells. This solute exchange allows for turgor interactions and epidermal ‘back pressure’—accessed through *Constraint Relaxation* (*CRR*) parameters (Figure 2c)—and it accommodates the viscoelastic properties of the guard cell wall (Jezek et al., 2019). The OnGuard platform also assumes a single endomembrane compartment, referred to as the vacuole. This simplification avoids the need to define additional sets of transporters and their parameters for other endomembrane compartments, and it accords with the role of the vacuole as the primary endomembrane compartment responsible for holding the bulk of the osmotically active solute in the guard cell. In general, our knowledge of transport between the cytosol and compartments such as the endoplasmic reticulum, mitochondria and chloroplasts remains insufficient to quantify their functions without introducing indetermination to OnGuard outputs. Nonetheless, the platform retains the capacity for users to add these compartments in the future, once sufficient experimental detail becomes available.

The *Physiology* and *Biophysics* modes give the user access to the populations of the plasma membrane and tonoplast ion channels, carriers and pumps through the *Transporters* page (Figure 2c). This facility enables manipulations that simulate null mutants and transporter overexpression by setting the corresponding population number to zero and to higher values, respectively. In each case, the user selects the transporter from the dropdown list and enters values in the *Number* entry box to the right of the transporter identifier. Note that the number of transporters specified is that of the single guard cell, not the density of transporters per unit area of membrane. Parameters defining the biophysical and intrinsic regulatory properties of the various transporters—*Ion Channel*, *Pump* and *Carrier*—are also accessible through the *Biophysics* mode.

The *Metabolism* page (Figure 2c) gives access to the concentrations of Mal and sugar, the latter labelled as sucrose (Suc) for expedience, as well as the characteristics of photosynthesis in the guard cells. A preview of the diurnal cycle of Mal and Suc synthesis and their breakdown in the cytosol is available with the *Preview* button, but note that the preview does not take account of solute transport between compartments and is intended as a guide only. In the *Biophysics mode*, this page includes the option change the concentrations of each solute in each of the three compartments, and to adjust the characteristics for guard cell photosynthesis and for the interconversion between Suc and Mal in the cytosol.

The *Water‐Use Efficiency* page (Figure 2c) allows the user to set parameters for stomatal pore and leaf geometries, and mesophyll carbon assimilation. Assimilation parameters are also accessible through a preview function that shows the *A*–*C*_i_ relations and the predicted assimilation characteristics over the 24 h cycle. The user has access also to the RWF to the leaf, as well as the environmental characteristics for pCO_2_ and RH (*w*_air_). Note that RWF is calculated as the effective evaporative surface of the xylem and surrounding mesophyll below the stoma divided by the cross‐sectional area of the stomatal pore. RWF values of 40 and above correspond to a well‐watered plant with unrestricted water flow to the leaf and its transfer over cell surfaces within the leaf surrounding the xylem for evaporation. Water restriction can be imposed by reducing RWF to values of 10 and below (Wang et al., 2017).

Finally, the *Biophysics* mode gives the user access to the *Constraint Relaxation* (*CRR*) page (Figure 2c), defining the parameters for ion and turgor ‘exchange’ with the surrounding epidermal cells, the subsumed viscoelastic properties of the guard cell wall, and their opposing action on stomatal aperture. The *Biophysics mode* also gives access to parameters defining the relationship between guard cell solute content, volume, turgor and aperture through the General parameter page (Figure 2c).

## 6 THE OUTPUTS OF OnGuard3e

OnGuard outputs take the form of real‐time kinetics much as would be recorded in gas exchange experiments of transpiration and carbon assimilation rates, and it connects these outputs with those of ion and water flux, cellular [Ca^2+^]_i_, pH and Mal contents, among others. The platform, therefore, yields an abundance of information connecting the whole‐plant behaviour with the characteristics of the guard cell, its physiology and the underpinning molecular events of transport and metabolism. Of course, interpreting the output of any simulation relies on interrogating the variables it generates and their interconnections. To understand how a system responds to physiological or experimental perturbation, the user must determine the origins of each output variable and how one variable is connected to another, for example following an external trigger. Such information is vital, as each simulation, and the parameter set used to construct it, may be seen as a hypothesis under test, to be validated, refined or discarded by comparison with experiment.

**Figure 5:**
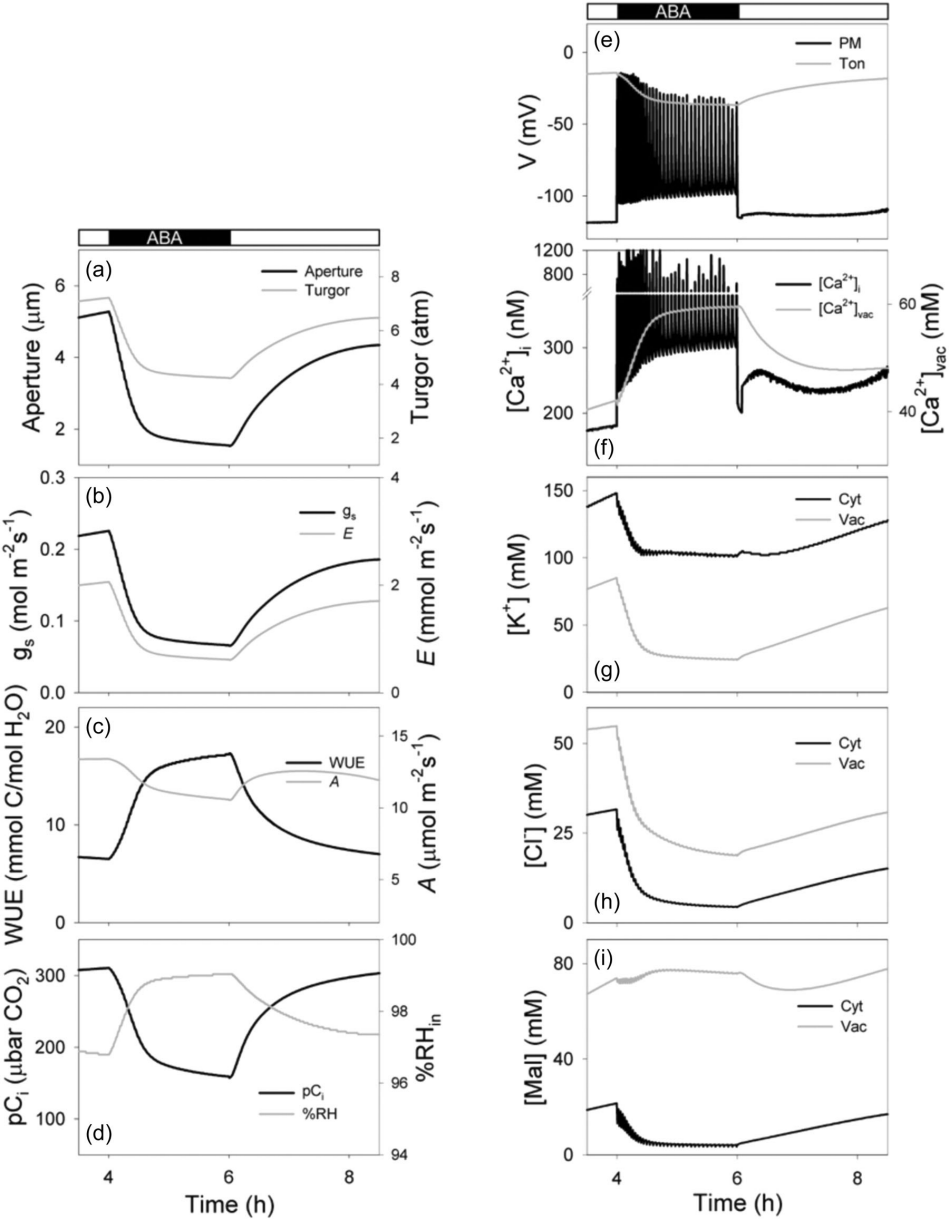
ABA‐evoked stomatal characteristics using the ‘hard‐wired’ OnGuard3e model. Selected outputs plotted from the data logged on running the ARAB‐wt‐ABA.ogb model with 1 μM ABA added over the period of 4–6 h in the light with 70% RH and 400 μbar CO_2_ in the atmosphere. Shown are (a) the stomatal aperture and guard cell turgor, (b) stomatal conductance, *g*_s_, and transpiration, *E*, (c) water‐use efficiency, and carbon assimilation, *A*, (d) the partial pressure of CO_2_, pC_i_, and %RH, in the leaf air space, (e) the plasma membrane and tonoplast voltages, (f) the cytosolic‐free Ca^2+^ concentration, [Ca^2+^]_i_, and total vacuolar Ca^2+^ concentration, and the cytosolic and vacuolar concentrations of K^+^ (g), Cl^−^ (h), and total Mal (i). ABA, abscisic acid; Mal, malate; RH, relative humidity.

We include here a selection of model outputs for the addition of ABA within the leaf (Figure 5). The core parameter sets provided (cf. ARAB‐wt.ogb) do not predefine higher‐order inputs—notably for the action of ABA—even though the dominant targets of the hormone are well known (Jezek & Blatt, 2017; Kollist et al., 2014; Lawson & Blatt, 2014; Willmer & Fricker, 1996). By leaving the targets of higher‐order inputs undefined, the user is free to address each pathway independently and may also assess the effects of assembling different combinations of targets. Nonetheless, we are frequently asked for a higher‐order model that includes ABA. With the OnGuard3e package, therefore, we now supply a file for a model defining the parameters for *Arabidopsis* that incorporates ABA action. Within the ARAB‐wt‐ABA.ogb model, ABA is ‘hard‐wired’ to a set of targets known to be affected by ABA and that also overlap with those resolved for pC_i_ action (Jezek et al., 2021). The model includes ABA as a user‐defined apoplastic solute with transporters at the plasma membrane and tonoplast. The primary targets for cytosolic ABA are the endomembrane and plasma membrane Ca^2+^‐ATPases along with the plasma membrane Ca^2+^ channel. These transporters impact on [Ca^2+^]_i_ and on pH_i_ for which ABA action is long‐established (Blatt & Armstrong, 1993; Hamilton et al., 2000; Jezek & Blatt, 2017; Wang et al., 2013). A full list of the relevant targets and parameter values for this model are included in Supporting Information: Appendix SA1.

Consistent with the literature (Assmann & Jegla, 2016; Jezek & Blatt, 2017), raising ABA in the apoplast to micromolar concentrations promotes a rapid decay in stomatal aperture, *E* and *g*_s_, even in the light, and a corresponding decline in *A* (Figure 5). Interrogating the simulation, as presented in the figure, shows that these declines are accompanied by a large decrease in guard cell turgor and volume, an increase and oscillations in [Ca^2+^]_i_, and by a substantial flux of K^+^, and Cl^−^ from the vacuole to the cytosol and apoplast across the plasma membrane. How are the connections made between ABA and guard cell transport? And how do these connections feedback to *g*_s_ and to *A* in the whole leaf?

We know that ABA promotes the gating of the plasma membrane Ca^2+^ channels and endomembrane Ca^2+^ release through the actions of ROS and nitric oxide (Garcia‐Mata et al., 2003; Grabov & Blatt, 1998; Hamilton et al., 2000; Kwak et al., 2003; Pei et al., 2000; Sokolovski et al., 2005), with their cumulative actions in promoting anion efflux while suppressing the activity of the KAT1‐type K^+^ channels (Jezek & Blatt, 2017). The repeated elevations in [Ca^2+^]_i_ suppress the plasma membrane H^+^‐ATPases and tonoplast H^+^‐PPase with corresponding impacts on the driving forces for transport at both membranes (Jezek & Blatt, 2017). The characteristics for enhanced Ca^2+^ release and its reduced elimination from the cytosol are encoded in the platform with ABA to overlap with those for the effects of CO_2_ (Jezek et al., 2021). In addition, the elevation of [Ca^2+^]_i_ in ABA suppresses the plasma membrane H^+^‐ATPase to accelerate closure. With these characteristics, the ARAB‐wt‐ABA.ogb parameter set reproduces stomatal behaviour that has been reported with ABA and, furthermore, it shows a reduction in pC_i_ and in *A* while reducing *E* and the raising the partial pressure of water vapour, *w*_p_, behind the stomatal pore.

A number of the predictions set out in Figure 5 are amenable to experimental testing. For example, the model predicts stomatal closure with ABA, even in the presence of reduced pC_i_ (Fischer et al., 1986), indicating a much more subtle balance of actions between water stress and CO_2_ availability (Raschke, 1976) than has been implied in recent studies of the underlying signal cascades (Chater et al., 2015; Schulze et al., 2021); it also shows an unexpected retention of Mal over Cl^−^ in the vacuole in the presence of ABA. Overall, these simulations establish a predominance in the connection of ABA signalling through guard cell Ca^2+^ transport. They suggest that endomembrane Ca^2+^ transport, especially, is vital to explaining this physiology and is sufficient to predict a dominance of the ABA stimulus over that of pC_i_ within the range typical for the gas in the inner leaf air spaces. Most important, the simulations highlight the feedback between the macroscopic processes of gas exchange and the microscopic regulatory network that operates in the guard cells.

## 7 CONCLUSIONS

Research on photosynthetic gas exchange and efforts to analyse stomatal behaviour through simulations have divided between two radically different approaches and scales. The physiology of the guard cell is exceptionally well‐defined and has been modelled successfully with quantitative mechanistic and kinetic detail at the molecular and cellular levels. By contrast, canopy‐level simulation of gas exchange has focused on stomatal aperture as a central parameter that is defined phenomenologically. Foliar gas exchange at this level is described with respect to water availability, atmospheric humidity, CO_2_, light and photosynthesis, but is devoid of the mechanics pertinent to the guard cell. Simply put, until now the absence of a detailed mechanistic platform for stomata has prevented efforts to introduce the developments from stomatal research at the cellular and subcellular levels in forward‐looking studies of the carbon and water cycles of the plant and the planet (Berry et al., 2010; Franks et al., 2017).

The OnGuard platform bridges this gap with an overarching approach that crosses scales while drawing explicitly on the molecular mechanics of transport, buffering and metabolism in the guard cell. The platform offers a new and unprecedented set of tools with which to explore the mechanics of foliar gas exchange within an overarching framework that operates seamlessly from the molecule to the plant canopy. These same tools offer opportunities in designing strategies for ‘reverse engineering’ of stomatal traits, assimilation and WUE. They are ideal as a basis for in silico ‘template trials’ to inform efforts in crop enhancement before their application in the field. Indeed, OnGuard modelling has proven important in guiding the recent success in engineering an ion channel native to the guard cell for enhanced WUE and biomass gains (Horaruang et al., 2022). The advances in synthetic biology toward modularising many biochemical and physiological processes (Papanatsiou et al., 2019; Yang et al., 2015; Zhu et al., 2010) means that such combinations of modelling and ‘template trials’ with laboratory and field experiments increasingly will become a central focus of research in the future.

Mechanistic platforms, such as OnGuard, are needed also to refocus large‐scale modelling efforts and better understand plant interactions with the environment. Berry et al. (2010) noted that the conclusions drawn from global‐scale modelling projects ‘depend on getting our plant physiology right’. If we are to improve on global‐scale models, it will be essential to avail ourselves of the mechanistic detail to hand for stomata. What makes the OnGuard platform so sucessful is its unique ability to model and predict temporal kinetics across a wide range of variable inputs with outputs that are immediately available for comparison with experiments in the field. Whether the focus is on whole‐plant ecophysiology or guard cell molecular biology, the OnGuard platform allows the user to explore and analyse transient behaviours as they arise, second by second, through the interacting elements of each model assembly. Rather than focusing on the steady‐state conductance of stomata, OnGuard describes stomatal physiology across timescales from fractions of a second to many hours and days as the plant trades soil water for CO_2_.

Even with the introduction of OnGuard3e, there remains much still to do. We encourage users to communicate with us and discuss ways of enhancing the platform to better serve the research community. A wealth of knowledge exists for guard cells of many species that has yet to be parameterised. Incorporating this knowledge within the OnGuard platform is sure to help expand the utility of the platform in guiding our understanding of stomata in the real world.

## Supporting information

Supporting information.

## Data Availability

The OnGuard3e platform and the model parameter sets as binary code described herein are freely available to academic users and may be downloaded from www.psrg.org.uk. The full list of entries to the core parameter set are also provided in the Supporting Information Appendix SA1.
