# Supplementary material for: OnGuard3e: A predictive, ecophysiology‐ready tool for gas exchange and photosynthesis research
Source: Plant Cell Environ. 2023 Jul 27;46(11):3644–58. doi: 10.1111/pce.14674 (PMC10946835; doi:10.1111/pce.14674)
Supplement: Supplementary file 1 — Supporting information. [file PCE-46-3644-s001.docx]

**Supplementary Information Appendix SA1**

Summary of Guard Cell Model: 'RCA5-wt-ABA'

Total cell volume = 0.346845 pL; vacuolar fraction = 85.1475%

Stomatal Aperture Parameters:

SA:P 'm'= 0.8 atm/µm; SA:P 'n'= 3 atm; SA:V 'R'= 0.05 pL/µm SA:V 'S'= 0.3 pL

Pore Length: 8 µm; Pore Depth: 15 µm

Cytosolic Protein Buffering: [Pr] = 0.126741 mM, pIso = 6.8, ap = -71

Cytosolic Calcium Buffering: [Bu] = 0.280203 mM, K = 3e-06 M # Ca Sites = 10

Temperature: Leaf: 25∞C; Air: 25∞C

Current 'time' in model = 00:00:00.00

*** Compartmental Solutions (/mM)

=================================

Apoplast Cytosol Vacuole

pH 6.500000 7.674381 5.374560

K 10.00000 94.09638 19.84146

Ca 1.000000 0.011318 42.25737

Cl 12.00000 14.49118 68.72348

Suc 0.010000 0.020571 0.002732

MH2 3.23e-07 1.67e-07 0.155459

MH 7.94e-06 0.000061 0.285874

M 0.009992 1.151313 26.96096

HCO3 0.022749 0.339892 0.001704

CO2 0.016166 0.016166 0.016166

ABA 1.00e-09 9.99e-10 1.00e-09

Malates 0.010000 1.151374 27.40230

*** Plasma Membrane Transporters

================================

PM K-in Channel [3000 units] (Inward-Rectifying GHK Channel)

---------------

#'K' G/Gmax = 9 pOhms

2-State Voltage Gate: VΩ = -185 mV,

Zg = +1.8

Light-Sensitive: NO!

Ligand-Gates:

Ca-inhibited (cytosol): Kd= 3.3e-07, Hill= 4;

H-activated (cytosol): Kd= 6e-08, Hill= 2;

H-activated (apoplast): Kd= 1e-07, Hill= 1;

PM K-out Channel [240 units] (Outward-Rectifying GHK Channel)

----------------

#'K' G/Gmax = 20 pOhms

VΩ = +1 ◊ F/RT ◊ ln([K]apo/10mM)

Zg = +2

Light-Sensitive: NO!

Ligand-Gates:

H-inhibited (cytosol): Kd= 3e-08, Hill= 2;

HCO3-activated (cytosol): Kd= 0.0003, Hill= 2;

R-Type Anion Channel [1000 units] (Outward-Rectifying GHK Channel)

--------------------

#'Cl' G/Gmax = 3.4 pOhms

#'M' G/Gmax = 2 pOhms

VΩ = +1 ◊ F/RT ◊ ln(1e-06mM/[H]cyt)

Zg = -2

Light-Sensitive: NO!

Ligand-Gates:

Ca-activated (cytosol): Kd= 6e-07, Hill= 4;

H-activated (cytosol): Kd= 3e-08, Hill= 3;

HCO3-activated (cytosol): Kd= 0.0003, Hill= 3;

V-Gated Ca-IN [12 units] (Inward-Rectifying GHK Channel)

-------------

#'Ca' G/Gmax = 12 pOhms

VΩ = +0.5 ◊ F/RT ◊ ln(2e-09mM/[Ca]cyt)

Zg = +1

Light-Sensitive: NO!

Ligand-Gates:

Ca-inhibited (cytosol): Kd= 5e-07, Hill= 5;

Anion VIC [300 units] (Outward-Rectifying Ohmic Channel)

---------

#'Cl' G/Gmax = 0.15 pOhms

#'M' G/Gmax = 0.07 pOhms

Voltage-Independent

Light-Sensitive: NO!

Ligand-Gates:

Ca-activated (cytosol): Kd= 6e-07, Hill= 4;

H-activated (cytosol): Kd= 4e-08, Hill= 2;

HCO3-activated (cytosol): Kd= 0.0003, Hill= 1;

H-ATPase [300000 units] (4-State 'Slayman' Pump)

--------

#'H' Stoichiometry = +1; binds at 4->1 (in) and 3->2 (ex);

K12 = 2000, K23 = 50000, K34 = 500, K41 = 2e+09,

K21 = 100, K32 = 1e+08, K43 = 10, K14 = 200;

Light-Sensitive: Yes: LΩ = 50 µEinsteins, Fmin = 5%

Ligand-Gates:

Ca-inhibited (cytosol): Kd= 2.5e-07, Hill= 3;

H:Cl Symport [50000 units] (4-State 'Slayman' Pump)

------------

#'H' Stoichiometry = +2; binds at 4->1 (in) and 3->2 (ex);

#'Cl' Stoichiometry = +1; binds at 4->1 (in) and 4->3 (ex);

K12 = 1000, K23 = 100, K34 = 50000, K41 = 1e+21,

K21 = 50, K32 = 1e+21, K43 = 100000, K14 = 100;

Light-Sensitive: NO!

Ligand-Gates:

<none>

H:K Symport [16000 units] (4-State 'Slayman' Pump)

-----------

#'H' Stoichiometry = +1; binds at 4->1 (in) and 3->2 (ex);

#'K' Stoichiometry = +1; binds at 4->1 (in) and 4->3 (ex);

K12 = 2, K23 = 10000, K34 = 100000, K41 = 1e+14,

K21 = 0.4, K32 = 1e+12, K43 = 1e+10, K14 = 50;

Light-Sensitive: NO!

Ligand-Gates:

<none>

Ca-ATPase [60000 units] (4-State 'Slayman' Pump)

---------

#'Ca' Stoichiometry = +1; binds at 4->1 (in) and 3->2 (ex);

K12 = 2000, K23 = 10000, K34 = 500, K41 = 1e+15,

K21 = 2, K32 = 1e+07, K43 = 500, K14 = 1000;

Light-Sensitive: Yes: LΩ = 50 µEinsteins, Fmin = 50%

Ligand-Gates:

Ca-activated (cytosol): Kd= 5e-07, Hill= 2;

HCO3-inhibited (cytosol): Kd= 0.0003, Hill= 4;

ABA-inhibited (cytosol): Kd= 3e-07, Hill= 1;

HMal symp [40000 units] (Concentration-Driven SYMPORT)

---------

#'H' (Stoichiometry = -3)

#'M' (Stoichiometry = -1)

Fmax = 1e+20

Light-Sensitive: NO!

Ligand-Gates:

<none>

K leak [1 units] (Inward-Rectifying GHK Channel)

------

#'K' G/Gmax = 1 pOhms

Voltage-Independent

Light-Sensitive: NO!

Ligand-Gates:

<none>

ABA uniport [30000 units] (Concentration-Driven UNIPORT)

-----------

#'ABA' (Stoichiometry = +1)

Kappa = 1e-06; Fmax = 10

Light-Sensitive: NO!

Ligand-Gates:

<none>

Kout (ABA) [96 units] (Outward-Rectifying GHK Channel)

----------

#'K' G/Gmax = 20 pOhms

VΩ = +1 ◊ F/RT ◊ ln([K]apo/10mM)

Zg = +2

Light-Sensitive: NO!

Ligand-Gates:

H-inhibited (cytosol): Kd= 3e-08, Hill= 2;

ABA-activated (cytosol): Kd= 3e-07, Hill= 1;

Anion VIC (ABA) [90 units] (Inward-Rectifying Ohmic Channel)

---------------

#'Cl' G/Gmax = 0.15 pOhms

Voltage-Independent

Light-Sensitive: NO!

Ligand-Gates:

Ca-activated (cytosol): Kd= 6e-07, Hill= 4;

H-activated (cytosol): Kd= 4e-08, Hill= 2;

ABA-activated (cytosol): Kd= 3e-07, Hill= 1;

R-type (ABA) [300 units] (Outward-Rectifying GHK Channel)

------------

#'Cl' G/Gmax = 3.4 pOhms

#'M' G/Gmax = 2 pOhms

VΩ = +1 ◊ F/RT ◊ ln(1e-06mM/[H]cyt)

Zg = -2

Light-Sensitive: NO!

Ligand-Gates:

Ca-activated (cytosol): Kd= 6e-07, Hill= 4;

H-activated (cytosol): Kd= 3e-08, Hill= 3;

ABA-activated (cytosol): Kd= 3e-07, Hill= 1;

Ca-ABA [12 units] (Inward-Rectifying GHK Channel)

------

#'Ca' G/Gmax = 12 pOhms

VΩ = +0.5 ◊ F/RT ◊ ln(2e-09mM/[Ca]cyt)

+ +1 ◊ F/RT ◊ ln([ABA]cyt/0.001mM)

Zg = +1

Light-Sensitive: NO!

Ligand-Gates:

Ca-inhibited (cytosol): Kd= 5e-07, Hill= 5;

*** Tonoplast Transporters

==========================

TPK1 [300 units] (Inward-Rectifying Ohmic Channel)

----

#'K' G/Gmax = 90 pOhms

Voltage-Independent

Light-Sensitive: NO!

Ligand-Gates:

Ca-activated (cytosol): Kd= 3e-06, Hill= 1;

H-activated (cytosol): Kd= 3e-08, Hill= 3;

TPC1 [100 units] (Outward-Rectifying GHK Channel)

----

#'Ca' G/Gmax = 27 pOhms

#'K' G/Gmax = 14 pOhms

VΩ = +1 ◊ F/RT ◊ ln([Ca]vac/2mM)

Zg = +2

Light-Sensitive: NO!

Ligand-Gates:

Ca-activated (cytosol): Kd= 3e-05, Hill= 1;

H-activated (vacuole): Kd= 1e-06, Hill= 1;

FV K Channel [1600 units] (Inward-Rectifying GHK Channel)

------------

#'K' G/Gmax = 6 pOhms

2-State Voltage Gate: VΩ = -30 mV,

Zg = +1

Light-Sensitive: NO!

Ligand-Gates:

Ca-inhibited (cytosol): Kd= 2e-07, Hill= 1;

H-inhibited (cytosol): Kd= 4e-07, Hill= 1;

VCL [300 units] (Inward-Rectifying GHK Channel)

---

#'Cl' G/Gmax = 40 pOhms

#'M' G/Gmax = 10 pOhms

VΩ = +1 ◊ F/RT ◊ ln([H]vac/0.005mM)

Zg = -1

Light-Sensitive: NO!

Ligand-Gates:

Ca-activated (cytosol): Kd= 1e-06, Hill= 1;

Vacuole H-ATPase [400000 units] (4-State 'Slayman' Pump)

----------------

#'H' Stoichiometry = +2; binds at 4->1 (in) and 3->2 (ex);

K12 = 100, K23 = 1000, K34 = 0.5, K41 = 1e+18,

K21 = 10, K32 = 1e+08, K43 = 5, K14 = 10000;

Light-Sensitive: Yes: LΩ = 50 µEinsteins, Fmin = 10%

Ligand-Gates:

<none>

Vacuole H-PPase [1200000 units] (4-State 'Slayman' Pump)

---------------

#'H' Stoichiometry = +1; binds at 4->1 (in) and 3->2 (ex);

K12 = 1000, K23 = 1000, K34 = 1e+11, K41 = 3e+09,

K21 = 100, K32 = 5e+09, K43 = 1e+07, K14 = 10000;

Light-Sensitive: Yes: LΩ = 50 µEinsteins, Fmin = 10%

Ligand-Gates:

Ca-inhibited (cytosol): Kd= 1e-07, Hill= 1;

K-activated (cytosol): Kd= 0.05, Hill= 1;

Slaymanesque Ca Pump [800000 units] (4-State 'Slayman' Pump)

--------------------

#'Ca' Stoichiometry = +1; binds at 4->1 (in) and 3->2 (ex);

K12 = 3000, K23 = 1000, K34 = 1000, K41 = 1e+09,

K21 = 0.3, K32 = 10000, K43 = 10, K14 = 10000;

Light-Sensitive: Yes: LΩ = 50 µEinsteins, Fmin = 50%

Ligand-Gates:

Ca-activated (cytosol): Kd= 3.5e-07, Hill= 3;

HCO3-inhibited (cytosol): Kd= 0.0003, Hill= 4;

Ca-inhibited (vacuole): Kd= 0.04, Hill= 4;

ABA-inhibited (cytosol): Kd= 3e-07, Hill= 1;

Vac.CLC [120000 units] (4-State 'Slayman' Pump)

-------

#'H' Stoichiometry = +1; binds at 4->1 (in) and 3->2 (ex);

#'Cl' Stoichiometry = -2; binds at 1->4 (in) and 2->3 (ex);

K12 = 1000, K23 = 1e+09, K34 = 100, K41 = 1e+10,

K21 = 1000, K32 = 1e+09, K43 = 10, K14 = 1e+11;

Light-Sensitive: NO!

Ligand-Gates:

H-inhibited (cytosol): Kd= 5e-08, Hill= 2;

Tonoplast VCa [8 units] (Outward-Rectifying GHK Channel)

-------------

#'Ca' G/Gmax = 10 pOhms

VΩ = +1 ◊ F/RT ◊ ln(10mM/[Ca]vac)

+ +0.5 ◊ F/RT ◊ ln(0.5mM/[HCO3]cyt)

Zg = +4

Light-Sensitive: NO!

Ligand-Gates:

Ca-activated (cytosol): Kd= 5e-07, Hill= 4;

T-deactivation: switch= [Ca]cyt; Threshold= 0.001mM, TΩ= 100000ms, reset= 5%/0.0005mM

CAX [100000 units] (Concentration-Driven ANTIPORT)

---

#'H' (Stoichiometry = -3)

#'Ca' (Stoichiometry = +1)

Fmax = 1e+22

Light-Sensitive: NO!

Ligand-Gates:

Ca-activated (cytosol): Kd= 3e-06, Hill= 1;

H-inhibited (cytosol): Kd= 4e-08, Hill= 3;

ALMT-Mal [600 units] (Inward-Rectifying GHK Channel)

--------

#'M' G/Gmax = 6 pOhms

2-State Voltage Gate: VΩ = +0 mV,

Zg = -2

Light-Sensitive: NO!

Ligand-Gates:

H-inhibited (cytosol): Kd= 8.5e-08, Hill= 2;

Ca-activated (cytosol): Kd= 1e-06, Hill= 1;

NHX [20000 units] (Concentration-Driven ANTIPORT)

---

#'H' (Stoichiometry = -1)

#'K' (Stoichiometry = +1)

Fmax = 100000

Light-Sensitive: NO!

Ligand-Gates:

<none>

Vac MLC [0 units] (4-State 'Slayman' Pump)

-------

#'H' Stoichiometry = +1; binds at 4->1 (in) and 3->2 (ex);

#'M' Stoichiometry = -1; binds at 1->4 (in) and 2->3 (ex);

K12 = 50000, K23 = 2e+07, K34 = 100, K41 = 1e+10,

K21 = 1000, K32 = 1e+09, K43 = 10, K14 = 1e+11;

Light-Sensitive: NO!

Ligand-Gates:

H-inhibited (cytosol): Kd= 5e-08, Hill= 2;

ABA uniport [30000 units] (Concentration-Driven UNIPORT)

-----------

#'ABA' (Stoichiometry = +1)

Kappa = 1e-06; Fmax = 10

Light-Sensitive: NO!

Ligand-Gates:

<none>

*** METABOLISM

==============

Total Malate (apo/cyt/vac) = 0.01 1.15137 27.4023 mM

Total Sucrose (apo/cyt/vac) = 0.01 0.020571 0.00273155 mM

Photosynthesis:

Suc s-max = 10 fmol/h, LΩ = 50 µE

Mal s-max = 0 fmol/h, LΩ = 50 µE

Light Type: Total

Sucrose Sink:

R-max = 10 fmol/h, KΩ = 1 mM

Suc <-> Mal Conversion:

R-max = 5 fmol/h, KΩ(S) = 0.1 mM, KΩ(M) = 10 mM

Mid-point pH = 7.7, pH gradient = +100

'Q10' Temperature Coefficient: 2

*** PHOTOSYNTHESIS & WUE PARAMETERS

===================================

Stomata/mm≤ = 100

Stomatal Length/µm = 8; Depth = 15

Subepidermal Depth/µm: = 300; Empty Space = 50%

CO2 Assimilation, A = AL ◊ AC - Rd [ Where AL = {fL + Amax-v[(fL + Amax)≤-4T.fL.Amax]}/2T and AC = 1/[1 + Kc/(Cin-Cc)])]

Amax (µmol/m≤/s) = 20

f = 0.15; T = 0.9

Kc (/ppm) = 80; Cc (/ppm) = 8; Rd (µmol/m≤/s) = 2

System used for Ciso = Mott (p-site)

Wet Surface Area Coefficient (RWF) = 40

Mott Potential Attenuator (Divisor) = 24.466

*** Constraint Relaxation & Recovery

====================================

Use CRR? Yes

Solutes to include: K

Epidermal Cell Capacity (Amax, /fmol) = 14

Adjacent Wall Capacity (Smax, /fmol) = 0.5

Max Recovery Rate (/fmol/sec) = 0.006; (Order = 1)

Current Contents = 100.0%

Apply Turgor-Sensitivity? Yes

Attenuator Mid-Point (atm) = 7; Attenuator Gradient (/atm) = 2
